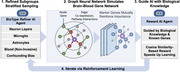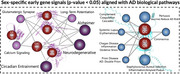# Non‐Invasive Blood‐Based Early Alzheimer’s Detection Using Sex‐Specific Brain‐Blood Graph Reinforcement Learning

**DOI:** 10.1002/alz70861_108586

**Published:** 2025-12-23

**Authors:** Claire Xu

**Affiliations:** ^1^ SFBay Association for Computing Machinery, San Francisco, CA USA

## Abstract

**Background:**

Alzheimer’s disease (AD) affects 1 in 9 individuals aged 65+ in the U.S., with females twice as likely as males. As AD pathology begins decades before symptoms appear, early detection and intervention are critical.

**Method:**

I developed NeuroPlasmaNet, an AI system that identifies early‐stage blood‐based biomarkers using graph neural networks (GNNs) to simulate the sex‐specific brain‐blood multi‐omics gene networks guided by neural pathology. Since AD is defined in the brain and detected in blood, I first constructed biotype‐stratified brain gene graphs based on cell types and layers, controlling for confounding biases such as age, APOE genotype, and education. I deployed reinforcement fine‐tuning with the cosine‐based similarity reward function to optimize each learning iteration and align predicted genes with AD biological relevancy. This approach revealed distinct male vs. female molecular signatures. I then refined non‐invasive blood‐based gene selection and identified NeuroPlasma12 gene panel. This brain‐first analysis grounded blood markers in AD neural pathology, avoiding systemic noise. I introduced the NeuroPlasma Score (NPS) to quantify the gene panel profile.

**Result:**

NeuroPlasmaNet achieved 92.03% accuracy (AUC=0.9410, *p* < 0.001) for early AD detection using NeuroPlasma12 gene panel (e.g., C1QB, TXNIP, TREM2, GFAP, PLCG2, CD163, CAMK1D, LRP10). I also identified potential therapeutic targets (e.g., GRIN2B, PLCG2, GRM5, CAMK1D), some novel and others published in *Nature* and *JAMA*. Beyond gene markers, the results highlight modifiable cognitive risk factors, such as substance use, infections, circadian disruption, and nutrition deficiencies, and promote personalized, preventive brain health strategies to the largely overlooked preclinical population.

**Conclusion:**

My study connects brain‐blood through sex‐specific gene graph networks, improving the blood markers precision by grounding it in neural pathology and avoiding noise from whole‐body blood circulation. Using **NeuroPlasmaNet**, an AI system incorporating cell type/layer stratification and biologically guided reinforcement learning, I identified **NeuroPlasma12 gene panel in blood transcriptomics**, validating my hypothesis that blood markers can detect early AD signals and support preventive brain health to **serve the 75% overlooked population**.